# Performance analysis of a solar desalination system operated by humidification–dehumidification technique

**DOI:** 10.1038/s41598-026-40700-6

**Published:** 2026-03-24

**Authors:** Abdalla Gomaa, Ahmed E. Hassaneen, Hatem Ibrahim, Mahmoud Abdelmagied

**Affiliations:** 1 Present Address: Department of Refrigeration and Air-Conditioning Technology, Faculty of Technology and Education, Capital (Formerly Helwan) University, Cairo, Egypt; 2 Present Address: Department of Automotive Technology, Faculty of Technology and Education, Capital (Formerly Helwan) University, Cairo, Egypt

**Keywords:** Desalination, Humidification–dehumidification, Gain output ratio, Recovery ratio, Energy science and technology, Engineering, Environmental sciences

## Abstract

The humidification–dehumidification desalination system represents a thermal-based technology primarily intended for small-scale water desalination applications. It facilitates the production of distilled water from saline seawater at sub-boiling temperatures by employing low-grade heat sources, such as solar energy or waste heat, which are both readily available and characterized by low operational costs. This study aims to enhance the yield of distilled water from actual seawater (Suez Canal water) while simultaneously reducing production costs under the climatic conditions of Cairo, Egypt. To achieve this, an experimental, economic, and environmental evaluation was conducted on a closed-air open-water humidification–dehumidification desalination system, which is recognized for its superior energy efficiency. The pilot plant was constructed, and experimental trials were performed during the months of February and March. The investigation focused on assessing the effects of varying the seawater mass flow rate for a range of 0.39–0.63 kg/s and the air velocity during daytime operation hours. A constant flow of city water was supplied to the dehumidifier coil to condense the water vapor from the moist air. System performance was monitored from 9 AM to 5 PM daily. The findings indicate that the maximum distilled water production reached 17.04 kg/day at a seawater flow rate of 0.63 kg/s and an air velocity of 13.2 m/s. The estimated cost of distilled water production was approximately 0.017 $ per liter, while the CO_2_ emission was reduced by 6 tons annually for the same amount of distilled water.

## Introduction

Water resource management is considered an absolutely strategic priority in Egypt. As the per capita size of irrigated land is shrinking and unemployment is relatively high, national and local water management institutions are increasingly challenged to provide answers to the water crisis. Water resources in Egypt are confined to the withdrawal quota from the Nile water; the limited amount of rainfall; and the shallow groundwater reservoirs. Desalination of sea water has been practiced regularly for over 50 years and is a well-established means of water supply. Solar energy is one of the most important sources of renewable, clean, and inexpensive energy available in Egypt, in addition to the establishment of many new tourist cities near the shores of the seas, which need to have available large amounts of fresh water. There were many researchers who investigated experimentally the performance analysis of the humidification–dehumidification (*HDH*) desalination system.

Many researchers have been investigating the *HDH* desalination system, Tiwari and Kumar^1^ presented an experimental investigation of a solar-based *HDH* desalination system. The integration between double-ended open evacuated tube solar air heaters with a phase change material as thermal energy storage is used. The study was carried out at air flow rates of 100–200 kg/hr, at a constant saline water flow rate of 300 kg/hr. The use of the phase change material extended the system’s operation by 3 h after sunset, increasing freshwater yield by approximately 6.2–8.9%. A compact, solar-powered, two-stage *HDH* desalination system with a shared dehumidifier was presented by Yang et al.^2^. The seawater was heated using a 6 m^2^ compound parabolic solar collector. The system achieved a gain output ratio (*GOR*) of 0.84 and produced freshwater at a low cost ranging from 4.1 to 6.0 $/m^3^. Several studies were conducted to improve the freshwater production in solar *HDH* desalination systems. Mahmoudi and Valipour^3^, Mahmoudi et al.^4,5^ presented experimental studies on the performance of solar *HDH* desalination systems with a metal scrap-based porous media and parabolic dish collector. The results indicated that the optimal configuration achieved 0.975 porosity and produced 0.4 L/day. At a feed flow of 1.2 L/min and 5000 ppm salinity, the system achieved a gain output ratio of 0.24 and a specific energy consumption of 3.48 kWh/m^3^.

An experimental investigation of a solar desalination system using a porous activated carbon tube as a humidifier within an *HDH *unit in Suez City, Egypt (29.9669°N, 32.5499°E), during September and October was conducted by El-Said et al.^6^. The study showed that the maximum daily freshwater output reached 6.12 kg, the highest *GOR* was 1.24, and the freshwater production cost was estimated at 0.0138 $/L. A theoretical and experimental investigation of a solar *HDH* desalination system using a closed-air cycle was conducted by Mohamed et al.^7^. The results indicated that increasing the air flow rate enhances water productivity but reduces *GOR* and component efficiencies while raising energy consumption. Higher cooling water flow rates positively affected both productivity and *GOR*, reaching peak values of 6.32 kg/hr and 0.87, respectively, at 6 kg/min. The cost of producing one liter of potable water was estimated at 0.012 $. The experimental results closely matched theoretical predictions, confirming the reliability of the model.

Shalaby et al.^8^ presented the Hybrid Solar *HDH* system, which was tested over consecutive days during the summer at various water and air mass flow rates to desalinate highly saline water. The test results showed that the *HDH* system was unable to produce significant amounts of freshwater when the feed water temperature is below 60 °C. The system achieved optimal performance with a water mass flow rate of 0.11 kg/s and an air mass flow rate of 0.0004 kg/s. Under certain conditions, the hybrid solar *HDH* system can produce up to 72 kg/day of freshwater when using saline water at 85 °C and operating for 8 h per day. A four-stage cross-flow *HDH* solar desalination system study using direct contact dehumidifiers was proposed by Zhao et al.^9^. The system achieved a water yield of 34.1 kg/(m^3^ hr) and a pure water production cost of 3.86 $/ton, with a total estimated investment cost of about 6563 $ for the entire system, including 42 m^2^ of solar collectors.

Dehghani et al.^10^ presented an experimental study of brine recirculation in *HDH* desalination system with a direct contact dehumidifier. Their results presented that increasing the salinity of recirculation brine from 10 to 30% enhances the overall recovery ratio of the system from 66 to 86%. However, the *GOR* of the system slightly decreases from about 0.65 to 0.45 by increasing the salinity from 10 to 30%. Several studies to evaluate the *HDH* desalination system were carried out by Esfanjani et al.^11–13^ with a parabolic dish collector in a new cylindrical–conical cavity receiver, a new brine recirculation method, and a brackish water flow rate. Their system achieved a maximum thermal efficiency of 45.49% and a peak daily water production of 0.966 L/day,^11^. The maximum freshwater production was 1.308 L/day^12^. With increasing the flow rate, the gain output ratio improved by 46.67%,^13^. Ahmed et al.^14^ presented an experimental study of the performance of *the* *HDH* desalination system with corrugated packing aluminum sheets in the humidifier. When the inlet cooling water temperature reduced from 28.5 to 17 °C, the distilled water production was increased from 10 to 15 L/hr, which was enhanced significantly. The total cost of fresh water produced from the system was about 0.01 $/L. Li et al.^15^ developed a novel, biodegradable lignocellulose-modified cotton fabric membrane for efficient oil–water separation and solar-driven freshwater generation, achieving over 99.9% oil-in-water separation efficiency.

The performance and productivity of a solar *HDH* desalination unit were investigated theoretically and experimentally by Hamed et al.^16^. The system productivity was tested during different operating times in two periods, the first from 9 AM to 5 PM, and the second after preheating from 1 to 5 PM. Results indicated that the best operating time for the *HDH* system was from 1 to 5 PM. The average productivity of the *HDH* unit was 11 L/day.m^2^ of a solar collector, with an estimated distillate water cost of 0.0578 $/L. An experimental investigation of the performance of a small-scale solar *HDH* desalination plant based on a new type of solar air heater with all-glass evacuated tubes was conducted by Li et al.^17^. The increase in the inlet sprayed water temperature in the pad humidifier from 9 to 27 °C improved the relative humidity of the outlet moist air from 89 to 97% and raised the outlet air temperature from 35 to 42 °C. A comparison study of single- and multi-stage *HDH* desalination systems was reported theoretically by Zamen et al.^18^. A two-stage pilot plant with an 80 m^2^ solar collector was constructed and tested in an arid region during summer and winter seasons. Test results revealed that summer production exceeded twice the winter production, reaching about 580 L/day, and fresh water productivity reached 7.25 L/day m^2^, which was 40% higher than that of the single-stage unit. Chang et al.^19^ presented an experimental investigation for working principles and operating conditions on the performance of a novel multi-effect solar desalination system based on the *HDH* process. The results showed that the *GOR* of the desalination plant can reach about 2.1, while the yield productivity was 54.2% at the flow rate of feed seawater of 1000 kg/hr, compared with a flow rate of feed water of 550 kg/hr.

Bakhtiarzadeh et al.^20^ presented a passive, solar-powered desalination system combining a solar heater, insulated evaporation chamber, and spiral condenser linked to a water-cooling tower for continuous freshwater production. The daily water output increases by 286.8% and 231.2%, with overnight production of 1.936 ± 0.0515 L. According to Sachidananda et al.^21^, the performance of a pyramidal solar still can be enhanced using two active techniques with a low-power fan and an ultrasonic humidifier. The ultrasonic-assisted configuration demonstrated the best results, achieving a maximum efficiency of 57% and an energy efficiency of 25.11%, along with a distillate yield of 0.33 L. The influence of packing material types, packing height, and configuration on the performance of an *HDH* desalination system was examined by Hatab et al.^22^. Revealing that the best operation, which occurred at the 0.6 m layer of cellulose kraft paper, had maximum inlet and cooling water flow rates of 6 kg/min and 16 kg/min, respectively. Under an inlet temperature of 70 °C and a closed-air cycle, the system produced 4.2 L/hr of fresh water with a *GOR* of 0.63.

Although a significant number of research studies have been performed on solar desalination systems using humidification and dehumidification techniques, there is still much that needs to be investigated, especially in the Suez Canal region. The Suez Canal region representas an economically and urbanly integrated region, balanced environmentally with an integrated international model for sustainable development that leads Egypt towards global competitiveness. So, the present study aims to analyze experimentally, economically, and environmentally the closed-air, open-water solar *HDH* desalination system. The effect of sea water (Suez Canal water) feeding flow rate, air flow rate, and solar radiation on the gain output ratio and yield are achieved in the present study, as well as cost analysis. This aspect has received limited attention in previous studies. The study uniquely examines how variations in Suez Canal water (salinity 37–41 ppt), feed flow rate, and air velocity influence real operating performance throughout daytime hours. Moreover, it provides new quantitative data on maximum freshwater yield and the associated low production cost, offering a fresh reference point for advancing small-scale, energy-efficient desalination technologies.

## Experimental test rig

**Fig. 1:**
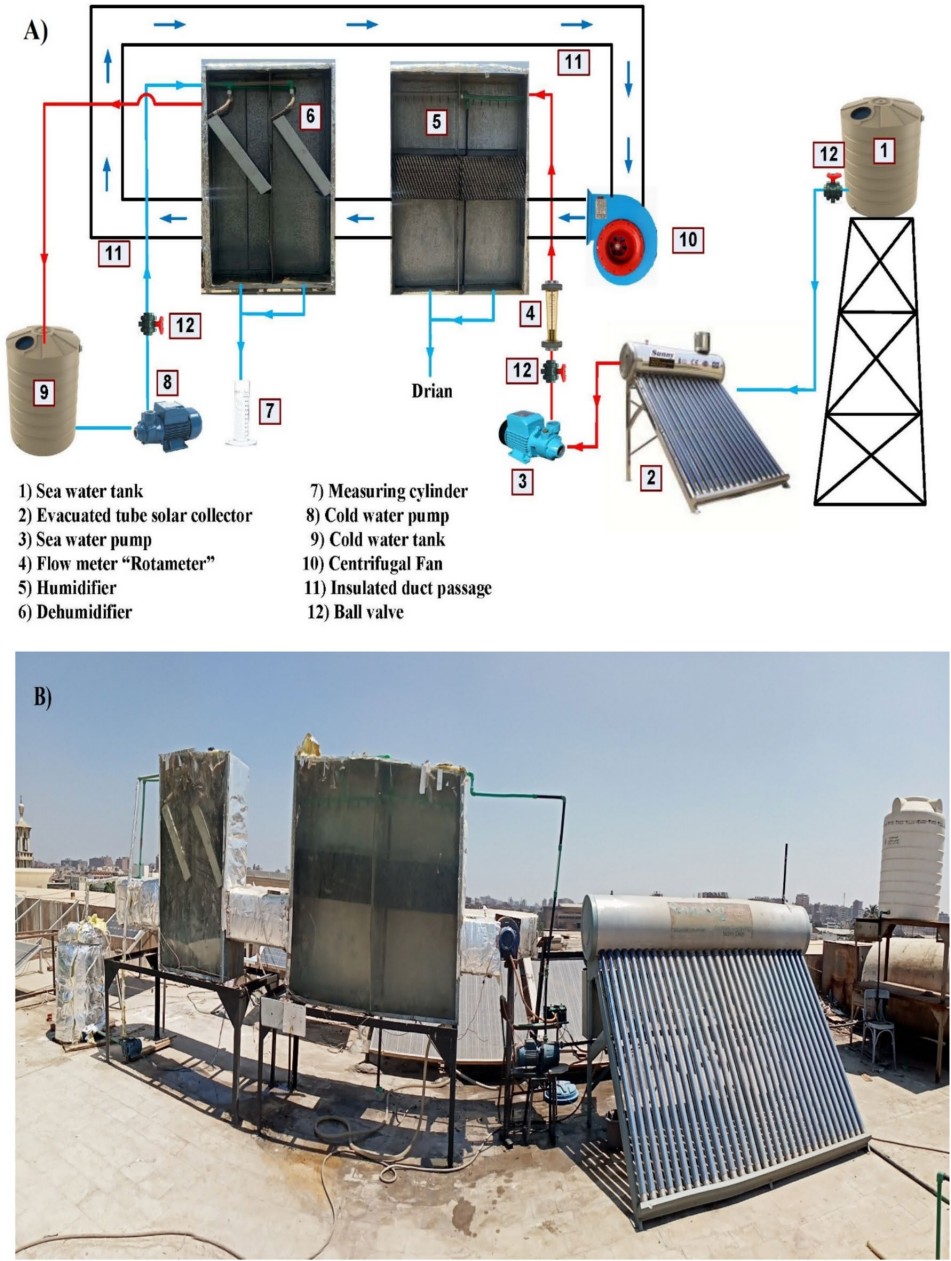
The experimental test rig. (**A**) Schematic diagram, and (**B**) Photograph of the test rig.

The schematic diagram and the photograph of the experimental test rig are illustrated in Fig. 1A,B, respectively. The experimental test rig consists of two main cycles. Open sea water flow cycle and closed air flow cycle. The first part of the water cycle is the evacuated tube solar collector, which consists of 30 vacuum tubes. A cylindrical stainless steel water tank insulated with 0.055 m of polyurethane foam and having a 300-L capacity is used to feed the humidifier with hot water through insulated tubes. The humidifier consists of a packing, humidifier body, and acrylic door in two stages. The specification of the packing used is illustrated in Table 1. The humidifier is made of 1.5 mm thick galvanized steel with a rectangular cross-sectional area of 0.4 × 1.6 m^2^ and a height of 2 m. The humidifier is insulated with fiberglass with a thickness of 1 inch and a density of 24 kg/m^3^. The base of the humidifier has a gradual slope to permit highly concentrated sea water to be blown down out of the humidifier. While the dehumidifier consists of a body, an acrylic door, and a condensing coil. The dehumidifier body is made of 1.5 mm thick galvanized steel with a rectangular cross-sectional area of 0.4 × 0.8 m^2^ and a height of 2 m. The condensing coil is made from a finned copper tube of 20 m length with aluminum louvered fins (12 fins/inch). The lower end of the dehumidifier consists of a cone-shaped basin formed from galvanized steel for collecting condensate distilled water. Three centrifugal pumps are used to circulate the water in the pipeline of the cold and hot water cycle. The sea water pump flow rate ranged from 0.39 to 0.63 kg/s. Ball valves are used to separate each part of the sea water loop from the system when it is necessary. A centrifugal fan is used to circulate the air through the closed insulated duct loop between the humidifier and dehumidifier. The sea water storage tank having a volume of 1 m^3^ is constructed 2 m above the ground, which helps to maintain a constant water flow rate. Sea water flows from the storage tank to the evacuated tube solar collector, where it is heated by solar energy up to 80 °C. After the solar collector heated the sea water in the tank, the water is pumped to the humidifier, where it is sprayed over the packing material using nozzles to facilitate evaporation. The moist air is then carried by a centrifugal fan through a closed duct toward the dehumidifier. The centrifugal fan transported the moist air at velocities ranging from 10.2 to 13.2 m/s. Meanwhile, the highly saline water collects at the bottom of the humidifier and is regularly discharged. The moisture-laden air passes into the dehumidifier, where it flows over cooling coils supplied with city water, which is causing the water vapor to condense. Finally, the condensed distilled water is collected at the bottom of the dehumidifier in a measuring cylinder. The geometric characteristic of the evacuated tube solar collector is illustrated in Table 2.

**Table 1:** Humidifier packing material specification.

Material	Self- extinguishing PVC
Operating temperature (°C)	60:75
Size (m)	0.3 × 0.3 × 1.6
Thickness before forming (micron)	270
Heat transfer surface area (m^2^/m^3^)	248
Void (%)	97
Cross corrugated flutes—angle (°)	60

**Table 2:** The geometric characteristic of the evacuated tube solar collector.

Specification	Dimensions/Material	Units
Collector dimensions (*LxW*)	1.8 × 2.5	m^2^
No. of evacuated tubes	30	–
Collector aperture area (*A*_*c*_)	3.78	m^2^
Glass material	Borosilicate glass	–
Glass tube outer / inner diameter	58/51	mm
Glass tube length	1800	mm
Glass transmittance	0.9	–
Glass absorptance	0.93	–
Emission factor	0.075	–
Manifold material	Stainless Steel 316L	–
Manifold diameter/ length	450/2500	mm

### Uncertainty analysis

The effect of experimental errors on the accuracy of the obtained results was evaluated through an uncertainty analysis following the methodology proposed by Alaian^23^. Considering the uncertainties in the independent variables (x_1_, x_2_, x_3_,…,x_n_), the corresponding uncertainties in the dependent results (*ψ*_R_) were determined using the root-sum-square method, which can be given as;

1 $$\psi_{R} = \sqrt {\left( {\frac{\partial R}{{\partial x_{1} }}} \right)^{2} \psi_{1}^{2} + \left( {\frac{\partial R}{{\partial x_{2} }}} \right)^{2} \psi_{2}^{2} + ......... + \left( {\frac{\partial R}{{\partial x_{n} }}} \right)^{2} \psi_{n}^{2} }$$

The uncertainty values of thermocouples, solar power meter, anemometer, measuring cylinder and calculated productivity were ± 0.2, ± 1.6, ± 0.06, ± 2.88 and ± 0.01 respectively.

## Data reduction

A comprehensive energy balance is now provided, including the solar energy input, the thermal storage tank, the latent heat gain in the humidifier, and the heat rejection process in the dehumidifier. The governing energy balance equations for each subsystem have been explicitly presented.

### Dehumidifier (condenser)

Energy and mass balance of the dehumidifier as follow:

2 $$Q_{in} = Q_{out} + Q_{loss}$$

3 $$Q_{in} = \dot{m}_{a} H_{a,in} + \dot{m}_{w} CP_{w} T_{w,in}$$

4 $$Q_{out} = \dot{m}_{a} H_{a,out} + \dot{m}_{w} CP_{w} T_{w,out}$$

5 $$Q_{loss} = A_{d} U_{d} \left( {T_{avg,d} - T_{\infty } } \right)$$

6 $$\dot{m}_{a}^{{}} (H_{a,in} - H_{a,out} ) + \dot{m}_{w}^{{}} CP_{w} (T_{w,in} - T_{w,out} ) = AU(T_{avg} - T_{\infty } )$$

The flow rate of the distillate water;

7 $$\dot{m}_{dw} = \dot{m}_{a} \left( {\omega_{in} - \omega_{out} } \right)$$

### Humidifier (evaporator)

Energy and mass balance of the humidifier as follow:

8 $$Q_{in} = Q_{out} + Q_{loss}$$

9 $$Q_{in} = \dot{m}_{a} H_{a,in} + \dot{m}_{w} CP_{w} T_{w,in}$$

10 $$Q_{out} = \dot{m}_{a} H_{a,out} + \dot{m}_{w} CP_{w} T_{w,out}$$

11 $$Q_{loss} = A_{h} U_{h} \left( {T_{avg,h} - T_{\infty } } \right)$$

12 $$\dot{m}_{a} \left( {H_{a,in} - H_{a,out} } \right) + \dot{m}_{w} CP_{w} \left( {T_{w,in} - T_{w,out} } \right) = A_{h} U_{h} \left( {T_{avg,h} - T_{\infty } } \right)$$

where;

13 $$T_{avg,d} = T_{avg,h} = \frac{{T_{a,d,in} - T_{w,d,out} }}{2}$$

14 $$T_{a,d,in} = T_{a,h,out} ,\;T_{a,d,out} = T_{a,h,in} ,\;H_{a,d,in} = H_{a,h,out} ,\;H_{a,d,out} = H_{a,h,in}$$

The thermal efficiency of the desalination system gain output ratio (*GOR*) is defined as the ratio of latent heat of vaporization of distilled water total amount of heat utilized to produce it^24^;

15 $$GOR = 100 \times \left( {\frac{{h_{dw} \times h_{fg} }}{{\dot{Q}_{in} + P_{p} }}} \right)$$

where *h*_*fg*_ is the latent heat of vaporization of water at water temperature in humidifier unit calculated according to Alaian et al.^23^;

16 $$h_{fg} = 2501.897 - 2.407T + 1.192 \times 10^{ - 3} T^{2} - 1.586 \times 10^{ - 5} T^{3}$$

The gain output ratio (*GOR*) is a performance indicator used to evaluate the thermal efficiency of thermal desalination systems, including humidification–dehumidification (*HDH*) systems. While overall gain output ratio (*GOR*_overall_) is defined as the ratio of the latent heat of the total produced fresh water to the total thermal energy input to the whole system per day.

The recovery ratio (*RR*) is defined as the ratio between the distilled water produced and the inlet sea water to the cycle and is calculated as the following^25^;

17 $$RR = \frac{{\dot{m}_{dw} }}{{\dot{m}_{sw} }}$$

The mass flow rate ratio (*MR*) is defined as the inlet sea water mass flow rate to the dry air mass flow rate circulated in the cycle, which is calculated according to Zubair et al.^24^;

18 $$MR = \frac{{\dot{m}_{sw} }}{{\dot{m}_{a} }}$$

19 $${\mathrm{where}},\;\dot{m}_{a} = \rho AV$$

The collector efficiency *η*_*c*_ is calculated;

20 $$\eta_{c} = \frac{{\dot{Q}_{in} }}{{I_{rad} \times A_{c} }}$$

21 $${\mathrm{where}},\;\dot{Q}_{in} = \dot{m}_{w} Cp_{w} \left( {T_{out} - T_{in} } \right)$$

## Results and discussion

The performance characteristics of the humidification–dehumidification solar desalination system were presented involving productivity, total productivity, gain output ratio, mass flow rate ratio, and recovery ratio at different key design parameters of daytime, saline water mass flow rate, and air velocity. In particular, the salinity of the Mediterranean Sea water ranges from 38 to 39 parts per thousand (ppt), while the salinity of the Red Sea water ranges from 36 to 41 ppt. The salinity of the Suez Canal water at Suez City ranges between 37 and 41 ppt because it is influenced by the waters of the Red Sea and the Gulf of Suez streams. This salinity is higher than the global average salinity of ocean water (which is approximately 35 ppt).

The solar humidification and dehumidification plant (test rig) is built in Cairo, Egypt. (Latitude 30.10°N, Longitude 31.29°E). All the experimental runs through this study used the Suez Canal water with measurable salinity ranging from 36.9 to 37.75 ppt. The experimental results were obtained through a matrix of experimental runs, in which the air velocities vary over six levels in the range of 10.2–13.2 m/s (10.2, 10.8, 11.8, 12.1, 12.6, 13.2 m/s), and by varying the sea water flow rate over six values from 0.39 to 0.63 kg/s (0.39, 0.42, 0.45, 0.5, 0.56, 0.63 kg/s) for each air velocity. The total experimental runs equal 36 experiments; each experiment was recorded over a full working day, from 9:00 AM to 5:00 PM. A sample of the ambient (weather) conditions during one week in February 2025 is presented in Table 3, while the water is supplied to the condensing coil as city water with a temperature that varies from 18 to 19 °C depending upon the time of day.

**Table 3:** Sample of the ambient conditions of the current study through February 2025.

Date	day time	9 am	10 am	11am	12 pm	1 pm	2 pm	3 pm	4 pm
22/2/2025	T_o.a_ (°C)	20	25	25.3	30.6	28.8	27	26.6	23.8
	R.H_o.a_ (%)	58.7	58.3	55.4	53.4	52.1	48.5	44.8	42.5
23/2/2025	T_o.a_ (°C)	20.1	21.4	23	25.4	25.1	24.3	22.6	22
	R.H_o.a_ (%)	59.6	55.9	52.4	51.0	47.1	42.4	40.6	38.4
24/2/2025	T_o.a_ (°C)	18.6	20.6	23.7	30.4	27.6	25.1	22.7	20.2
	R.H_o.a_ (%)	58.5	53.9	52.4	51.4	48.7	44.5	43.8	39.8
25/2/2025	T_o.a_ (°C)	24.1	25.8	29.1	31.5	25.7	24.5	22.5	20.1
	R.H_o.a_ (%)	57.4	52.4	55.4	50.3	57.6	42.5	41.6	40.5
26/2/2025	T_o.a_ (°C)	22.8	25.3	27.1	28.2	27.2	25.6	23.7	22.2
	R.H_o.a_ (%)	55.2	51.5	48.3	44.5	42.2	39.5	38.5	37.6
27/2/2025	T_o.a_ (°C)	18	19.5	20.6	24.8	22.9	21.5	20.1	19.6
	R.H_o.a_ (%)	53.6	51.2	45.9	42	39.5	38	36.6	34.8

**Fig. 2:**
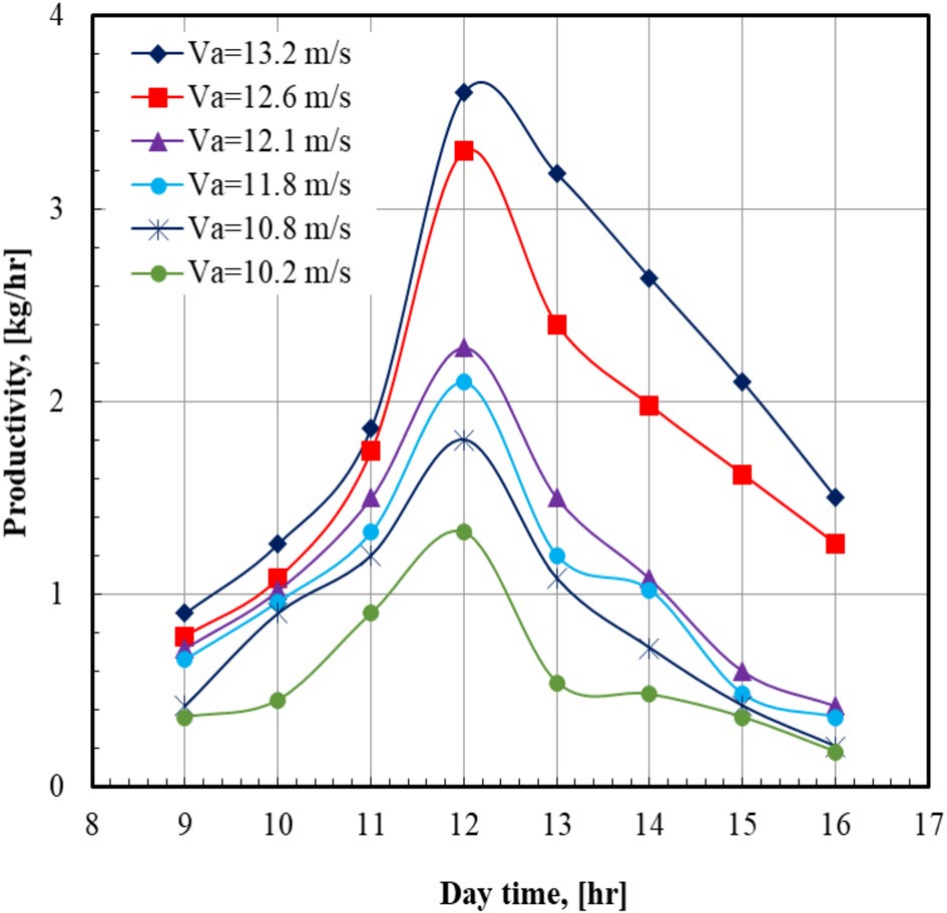
Productivity versus day time at m°sw = 0.63 kg/s.

The effect of daytime on the distilled water production (productivity) at various air velocities is shown in Fig. 2. The productivity increases from the morning and reaches the maximum values at noon, then decreases. The maximum value of the productivity occurred at an air velocity of 13.2 m/s at 12:00, which recorded 3.6 kg/hr. For the same daytime of 12:00, the productivity of the system at an air velocity of 13.2 m/s is higher than that of 10.2 m/s by 172.7%. This can be attributed to the increases in the air velocity that present a higher air volume flow rate, which carried more water vapor from the humidifier into the dehumidifier, which led to more distilled water being condensed.

**Fig. 3:**
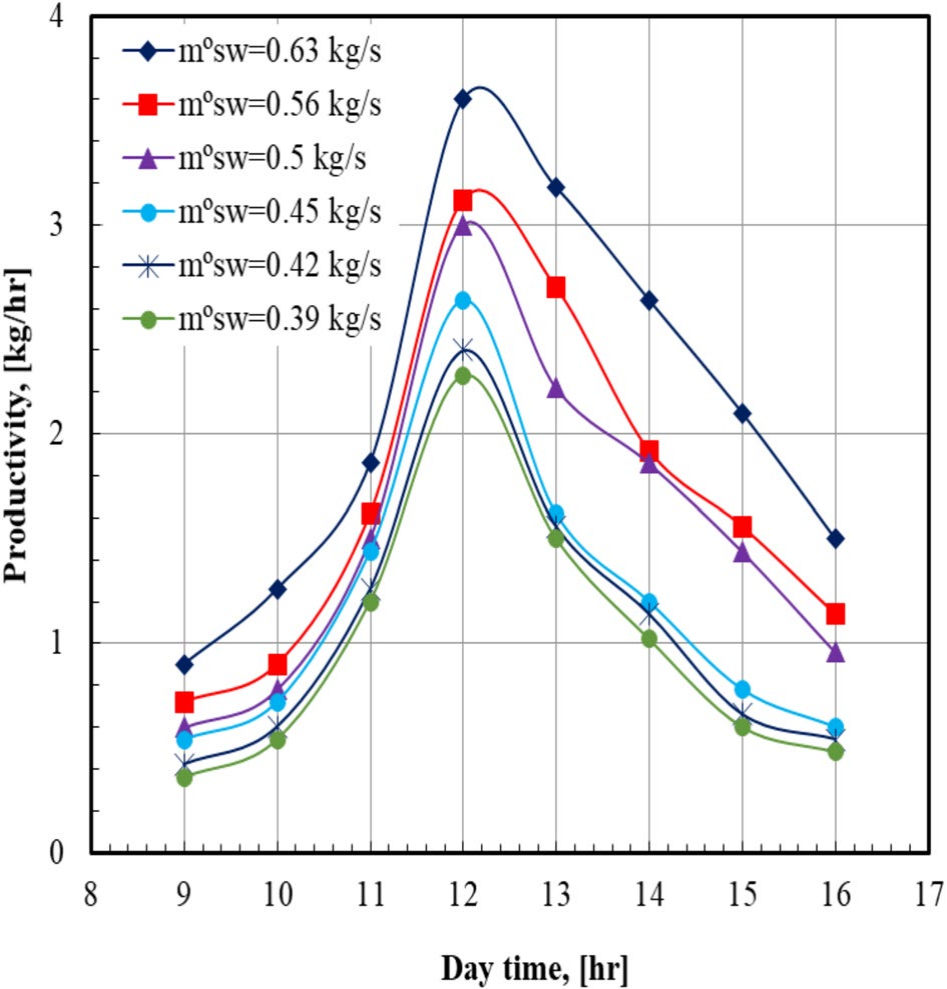
Productivity versus day time at Va = 13.2 m/s.

The results indicated that the productivity depended on the solar radiation; in the morning the heat absorbed is less and the rate of the evaporation from salty water is low. Furthermore, the air temperature and the solar radiation increased during the noon time, and hence the evaporation rate from salty water rose, which led to higher evaporation and production rates. This behavior is attributed to the high solar irradiance levels during this time. From afternoon to the evening hours, the solar intensity decreases, and the heat absorbed and the productivity decrease. For all values of sea water mass flow rate, it can be seen that the distilled water production (productivity) increases from the morning and reaches the maximum values at noon, then decreases. This can be clearly noticed from Fig. 3. This figure shows that the maximum value of the productivity occurred at sea water mass flow rate of 0.63 kg/s at 12:00, which is recorded as 3.6 kg/hr. At a certain daytime of 12 PM, the productivity of the system at sea water mass flow rate of 0.63 kg/s is higher than that of 0.39 kg/s by 57.9%.

**Fig. 4:**
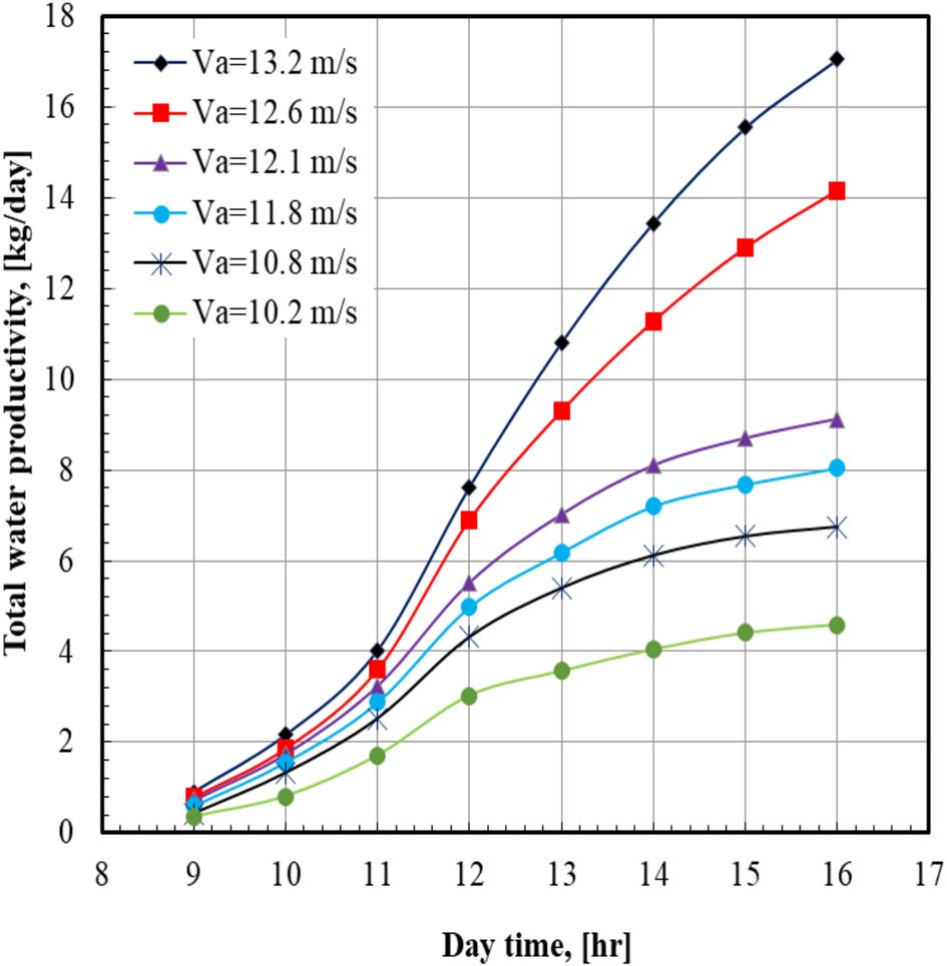
Total water productivity versus day time at m°sw = 0.63 kg/s.

**Fig. 5:**
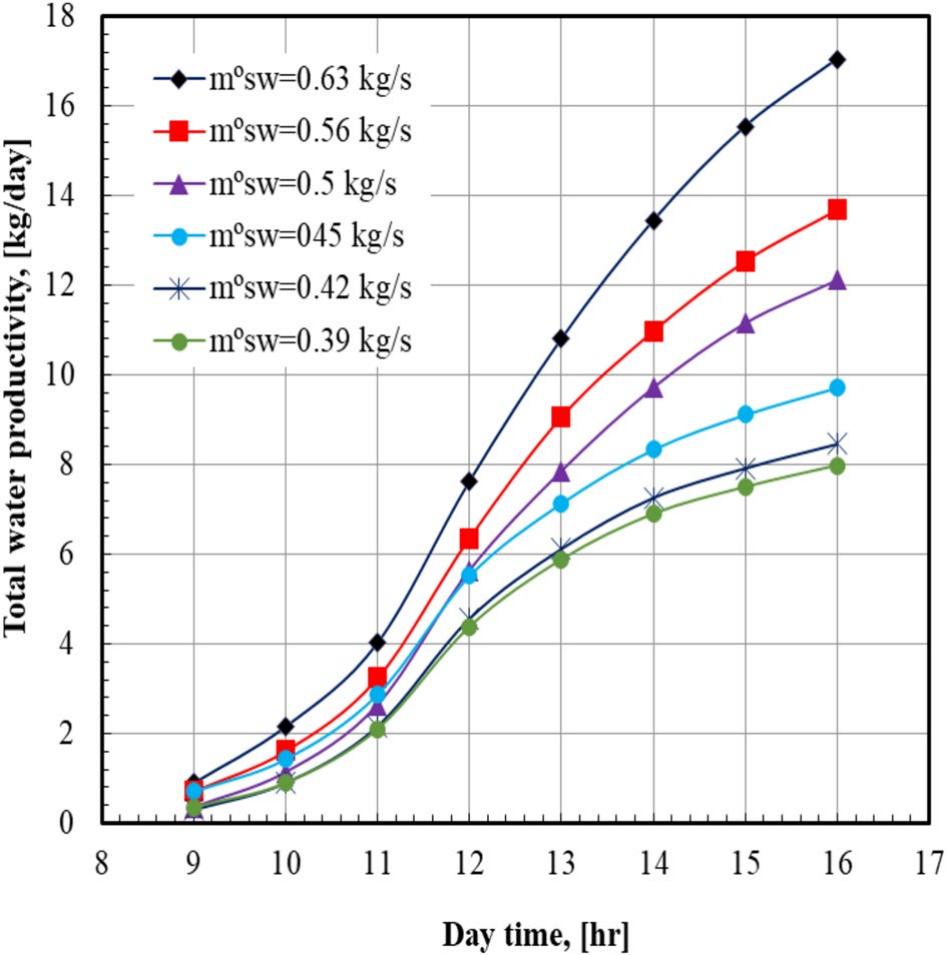
Total water productivity versus day time at Va = 13.2 m/s.

**Fig. 6:**
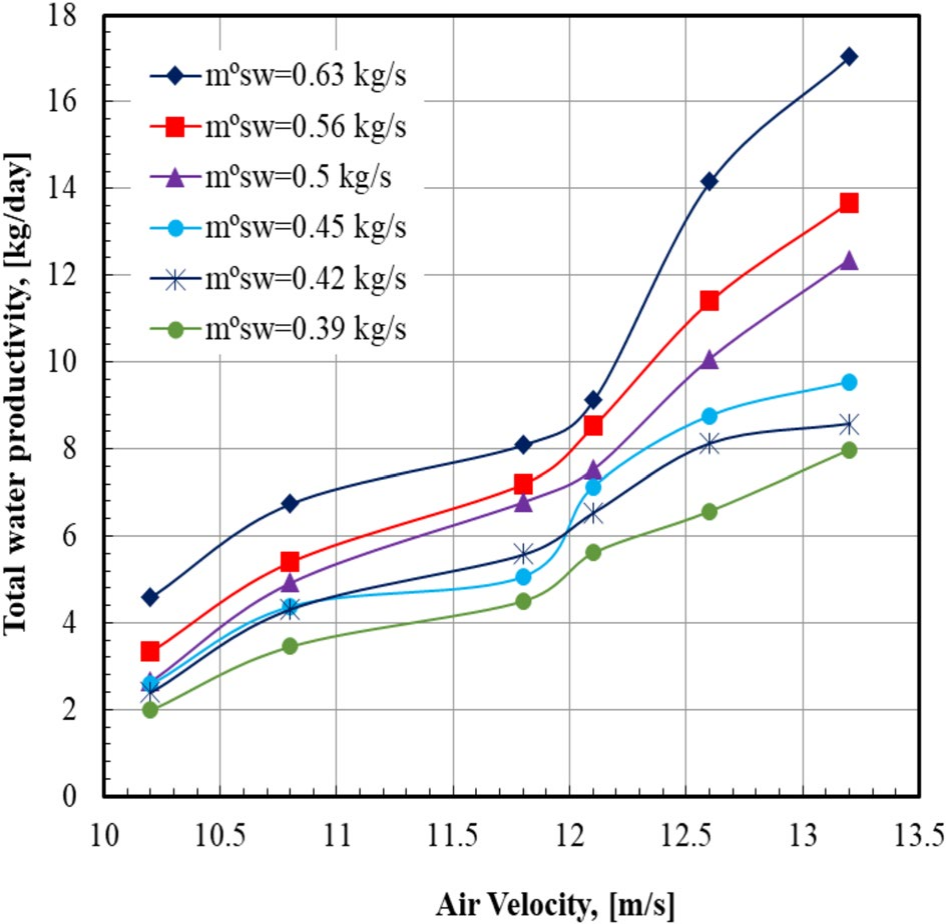
Total water productivity versus air velocity.

Figure 4 illustrates the total water productivity with the daytime at various air velocities. It is noted that the total water productivity increased from 4.59 to 17.04 kg/day when the moist air velocity increased from 10.2 to 13.2 m/s; this represents a percent increase of 271.24%. For all values of sea water mass flow rate, it can be seen that the total water productivity of distilled water increases from the morning until the distilled plant is switched off. It can also be noticed from Fig. 5 that the total water productivity increases by 113.5% from 7.98 to 17.04 kg/day when the sea water mass flow rate increases from 0.39 to 0.63 kg/s. For all values of sea water mass flow rate, it can be seen that the total water productivity of distilled water increases by increasing the air velocity. It can also be noticed from Fig. 6 that the maximum value of the total water productivity of distilled water (17.04 kg/hr) occurred at sea water mass flow rate of 0.63 kg/s with the air velocity = 13.2 m/s. At a certain value of air velocity, 13.2 m/s, the total water productivity of the system at sea water mass flow rate of 0.63 kg/s is higher than that of 0.39 kg/s by 113.5%.

**Fig. 7:**
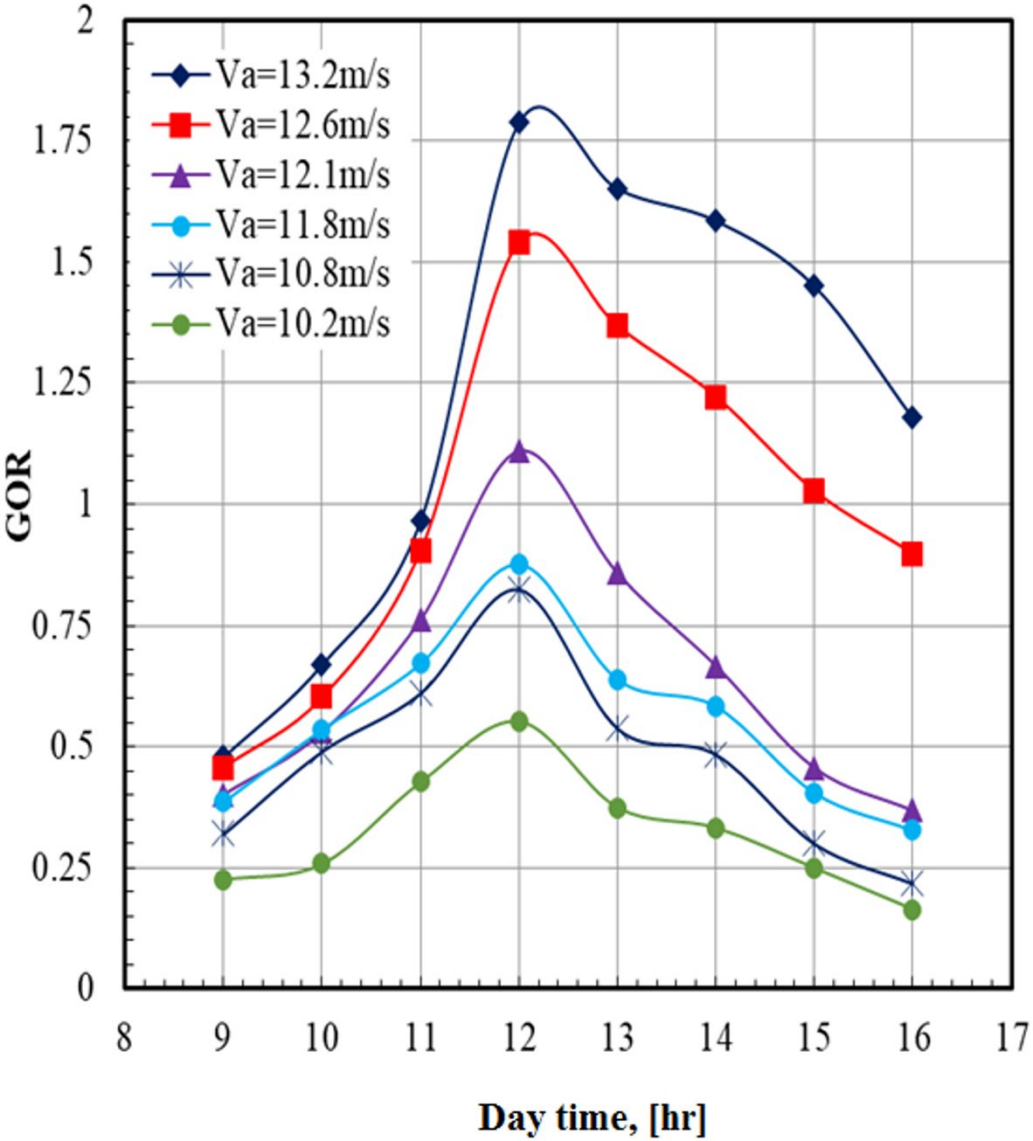
Gain output ratio versus day time at m°sw = 0.63 kg/s.

The effect of the air velocity on the gain output ratio (*GOR*) at the sea water mass flow rate of 0.63 kg/s at different times of day is illustrated in Fig. 7. The gain output ratio (*GOR*) increases from the morning and reaches the maximum values at noon, which reflects a maximum solar intensity, then decreases. The maximum value of the *GOR* (1.79) occurred at an air velocity of 13.2 m/s and 12 PM. At a certain daytime of 12 PM, the *GOR* of the system at an air velocity of 13.2 m/s is higher than that of 10.2 m/s by 225.45%. This can be referred to as the solar incident effect on the distilled water production according to the heat absorbed by the collector.

**Fig. 8:**
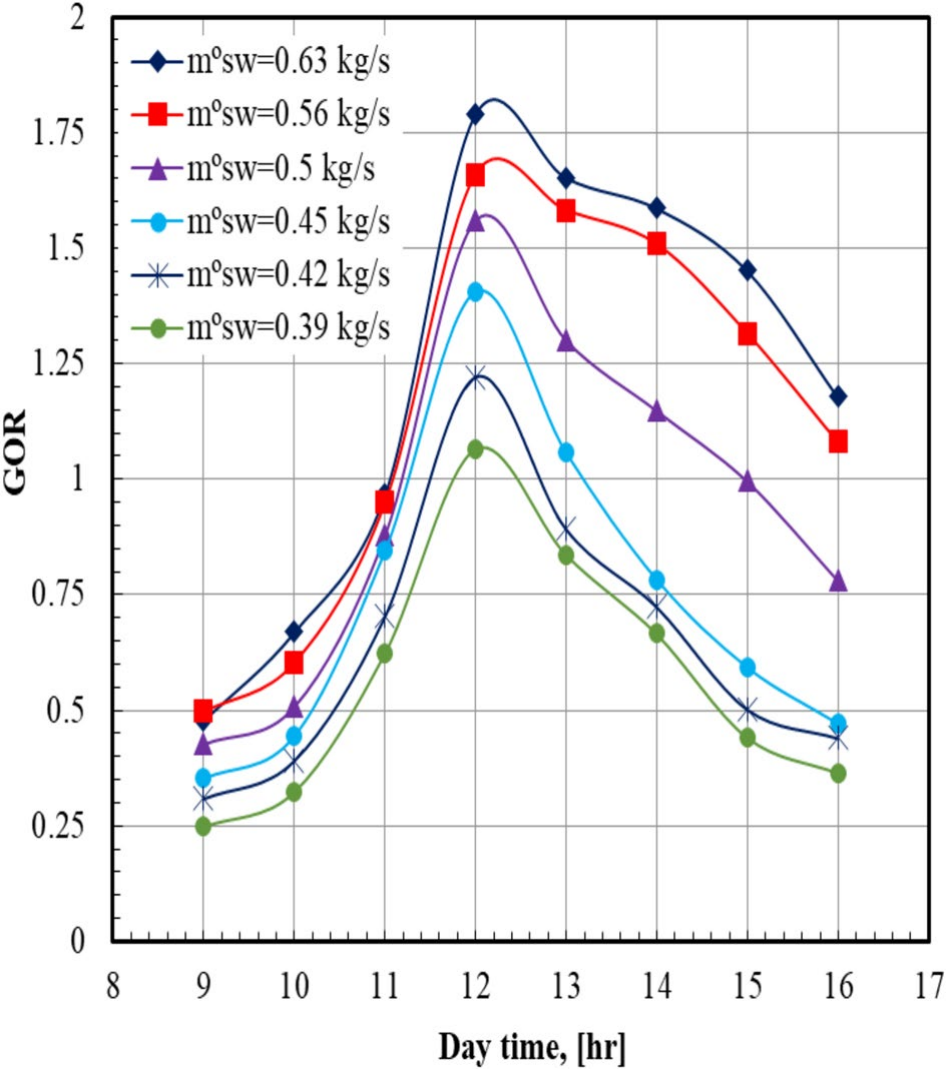
Gain output ratio versus day time at Va = 13.2 m/s.

The results indicated that the system productivity is strongly influenced by solar radiation throughout the day. In the morning, the absorbed heat is relatively low, leading to limited saltwater evaporation. As the air temperature and solar radiation increase toward noon, the evaporation rate rises significantly, resulting in higher distilled water production. This behavior is directly related to the elevated solar irradiance during midday. From the afternoon to evening hours, the decline in solar intensity reduces the absorbed heat and consequently decreases productivity. The effect of varying salty water mass flow rate on *GOR* over the daytime is illustrated in Fig. 8. For all values of sea water mass flow rate, it can be seen that the *GOR* increases from the morning and reaches the maximum values at noon, then decreases. The maximum value of the *GOR* (1.79) occurred at sea water mass flow rate of 0.63 kg/s and a daytime of 12 PM. At a certain daytime, the *GOR* of the system at sea water mass flow rate of 0.63 kg/s is higher than that of 0.39 kg/s by 68.9%.

**Fig. 9:**
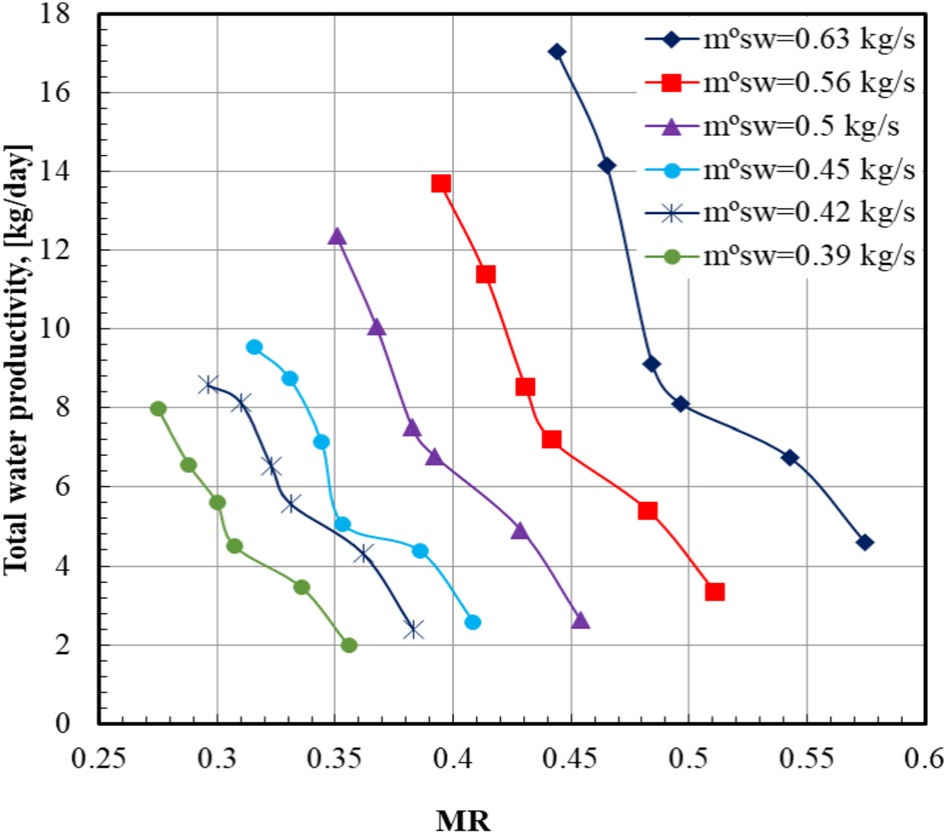
Total water productivity versus mass flow rate ratio at Va = 13.2 m/s.

Figure 9 illustrates the total water productivity with the mass flow rate ratio *MR* (water to air flow ratio) in the dehumidifier at various sea water mass flow rates. The total water productivity of distilled water decreases by increasing the *MR*. The maximum value (17.04) of the total water productivity occurred at sea water mass flow rate of 0.63 kg/s at *an* *MR* of 0.44. The reason for this phenomenon is the decreasing of the water temperature and the driving force of humidification in the humidifier. The productivity increases by increasing the moist air velocity; however, the mass flow rate ratio increases by decreasing the air velocity. The total distilled water productivity decreases from 17.04 kg/day to 4.59 kg/day, and the mass flow rate ratio increases from 0.44 to 0.57 at a feed seawater rate of 0.63 kg/s.

**Fig. 10:**
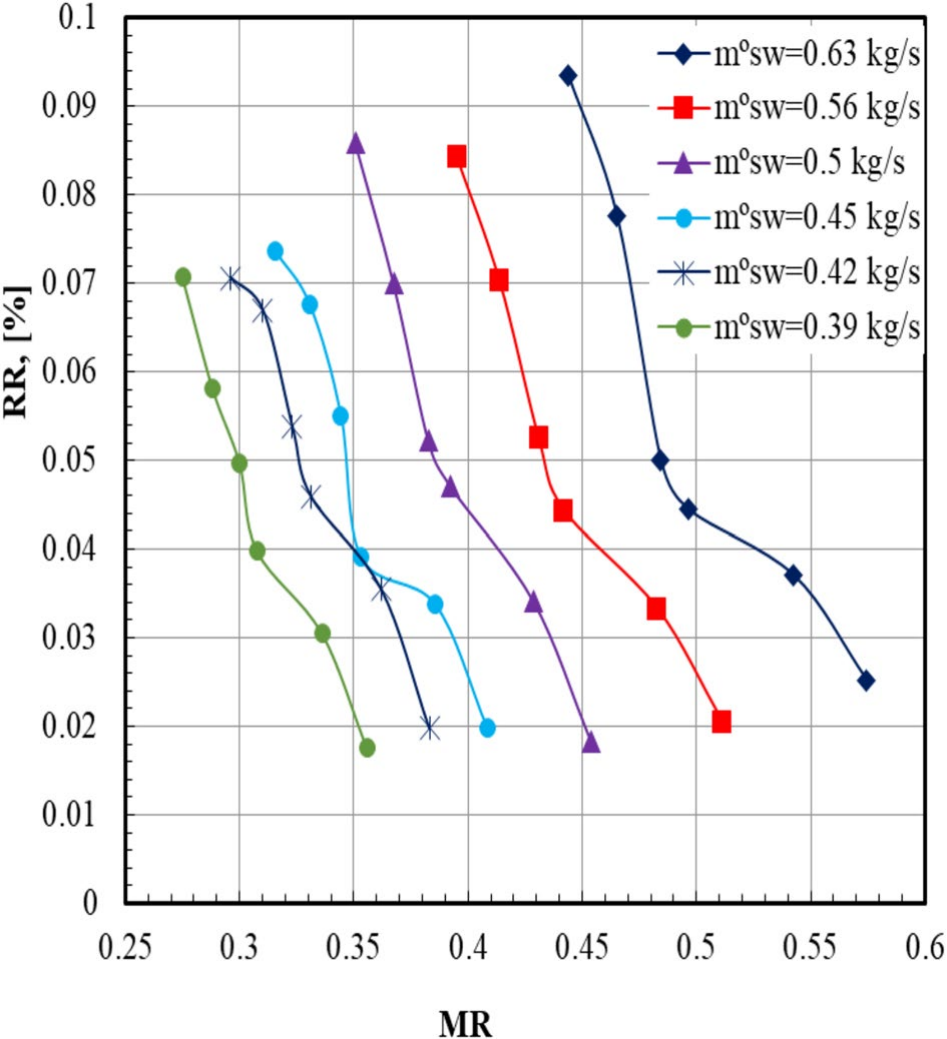
Recovery ratio versus mass flow rate ratio.

For all seawater mass flow rates, it can be seen that the recovery ratio (*RR*) decreases by increasing the *MR* in the dehumidifier, which is illustrated in Fig. 10. It can be noticed that the maximum value of the *RR* (0.093) occurs at sea water mass flow rate of 0.63 kg/s and *MR* = 0.44. The reason for this phenomenon is that the recovery ratio decreases by increasing the sea water mass flow rate; however, the mass flow rate ratio increases by increasing it. The recovery ratio decreases from 0.093 to 0.025% as the mass flow rate ratio increases from 0.44 to 0.57.

**Fig. 11:**
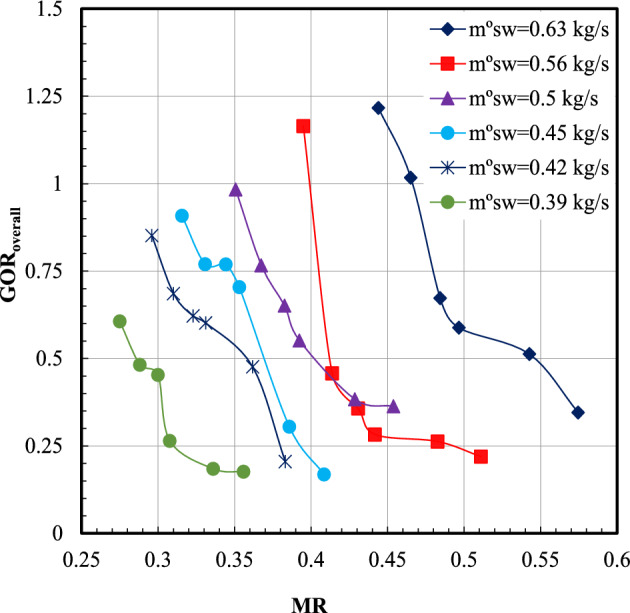
Gain output ratio overall versus mass flow rate ratio.

Figure 11 illustrates the *GOR*_overall_ with the *MR* at various sea water mass flow rates. The overall gain output ratio is the ratio of the latent heat of the total produced fresh water to the total thermal energy input to the whole system per day.

The *GOR*_overall_ decreases by increasing the *MR* in the dehumidifier. The maximum value of the *GOR*_overall_ occurred at sea water mass flow rate of 0.63 kg/s and *MR* of 0.44, which is recorded as 1.22. The *GOR*_overall_ decreases from 1.22 to 0.34 when the MR increases from 0.44 to 0.57. A sample of the results summary of the system outputs at different air velocities from February to March 2025 through six working weeks at a water mass flow rate of 0.63 kg/s is presented. The average of productivity, *GOR*_overall_, *RR*%, and *MR* over six days (one working week) divided by 6 to obtain the average parameter per day with air velocity is illustrated in Table 4.

**Table 4:** The system outputs at different air velocity through February and March 2025.

Week No	Date	v_air_ (m/s)	Average weekly productivity (kg/day)	*GOR*_overall_	*RR* (%)	*MR*
Week-1	15–20/Feb.	13.2	11.53	0.881	0.08	0.346
Week-2	22–27/Feb.	12.6	9.85	0.839	0.069	0.363
Week-3	1–6/Mar.	12.1	7.41	0.785	0.052	0.378
Week-4	8–13/Mar.	11.8	6.21	0.7	0.043	0.387
Week-5	15–20/Mar.	10.8	4.87	0.6	0.034	0.423
Week-6	22–27/Mar.	10.2	2.92	0.385	0.02	0.448

## Economic analysis

The economic analysis of the humidification–ehumidification desalination system is considered one of the most important factors used to evaluate the system’s efficiency and effectiveness through efforts to reduce the cost of producing desalinated water. *HDH* plant cost estimation for various components of the system is given in Table 5. The total fixed cost of the plant is about *F* = 1000$.

**Table 5:** Total fixed cost estimation for *HDH* plant.

*HDH*—Desalination plant cost	
Unit	Cost $
Solar collector 3 m^2^	410
Pumps and fans	50
Coil of heat exchanger	80
Packing materials	30
Body of *HDH* and ducts	200
Tanks and measurement cylinders	80
Piping system and nozzles	50
Support legs	20
Production cost (Sea water transport)	80
Total cost	1000 $

The capital recovery factor (*CRF*) is calculated according to Mahmoudi et al.^5^;

22 $$CRF = \frac{{i(1 + i)^{n} }}{{(1 + i)^{n} - 1}}$$

where *i* is the interest rate for bank, and it can be assumed 12%, and n is the life time of the system, where it can be assumed 10 years^7^.

The fixed annual cost (*FAC*)^5^;

23 $$FAC = F \times CRF$$

Sinking fund factor (*SFF*)^5^;

24 $$SFF = \frac{i}{{(1 + i)^{n} - 1}}$$

The salvage value of *HDH* desalination system (*S*) can be assumed 20% from the unit total cost^7^;

25 $$S = 0.2 \times F$$

The annual salvage value (*ASV*);

26 $$ASV = S \times SFF$$

The annual maintenance cost (*AMC*) can be assumed of 15% from the fixed annual cost;

27 $$AMC = 0.15 \times FAC$$

The total annual running cost (*AC*)

28 $$AC = FAC + AMC - ASV$$

The cost per liter production of distilled water (*CPL*) can be determined by the ratio between total annual cost (AC) and total annual productivity (*M*);

29 $$CPL = \frac{AC}{M}$$

The annual productivity of the *HDH* desalination system is found to be 9792 L/year. The daily period of running the desalination system is 8 h/day (from 9 AM to 5 PM). The *HDH* desalination system is assumed to operate 340 days/year, as Egypt is characterized by sunshine throughout the year^7^. The cost of producing one liter of desalinated water by the proposed system is 0.017 $/L. A comparison of the fresh water production rate and the cost of plant productivity for different types of thermal sources with the corresponding previous studies was presented in Table 6.

**Table 6:** Comparison of fresh water production rate and productivity cost with previous studies.

Lead researcher	Type of thermal source a desalination	Fresh water productivity (L/day)	Cost per liter (USD/L)
Current study	Evacuated tube collector	17.04	0.017
Mahmoudi et al.^5^	Parabolic dish collector	4.76	0.043
El-Said et al.^6^	Evacuated tube collector	6.12	0.0138
Hamed et al.^16^	Evacuated tube collector	22	0.0578
Mohamed et al.^7^	Evacuated tube collector	6.16 (L/hr)	0.012
Yang et al.^2^	Compound parabolic concentrator	29.5 (L/hr)	0.0041–0.006

## Environmental analysis

The environmental analysis to the system was performed based on the formulation for estimating carbon dioxide emissions reduction during the life time of the investigated desalination system^5^.

30 $$\Phi_{co2,\;En\;out} = \frac{{\left( {En_{output,\;annual} \times n} \right) \times 2}}{1000}$$

The amount of energy (electrical or fuel energy) saved due to using solar energy for obtaining the same amount of distilled water over a year for the lifetime of the plant, and the reduction of the estimated number of tons of CO_2_ emissions to the environment, are presented in Table 7.

**Table 7:** Environmental analysis results.

En_output,annual_ (kwh/year)	303.7
Co_2_ reduction over life time of the system (ton)	6

## Conclusion

The present study conducts an experimental, economic, and environmental evaluation of a closed-air, open-water *HDH* desalination system operating with real seawater (Suez Canal water) under the climatic conditions of Cairo. This work provides new insights into performance optimization, offers updated productivity and cost data, and contributes a reference for developing efficient small-capacity *HDH* desalination systems. The effect of the seawater mass flow rate for a range and air velocity for a range of 10.2–13.2 m/s with daytime on the system performance is tested. The following conclusions are summarized as;

- The productivity of the system at an air velocity of 13.2 m/s is higher than that of 10.2 m/s by 172.7% and 12 PM.
- The total water productivity increases by 271.24% from 4.59 to 17.04 kg/day when the air velocity increases from 10.2 to 13.2 m/s. Moreover, the total water productivity increases by 113.5% from 7.98 to 17.04 kg/day when the seawater mass flow rate increases from 0.39 to 0.63 kg/s.
- The maximum value of the *GOR* occurred at an air velocity of 13.2 m/s and a seawater mass flow rate of 0.63 kg/s at 12 PM.
- The *GOR* of the system at an air velocity of 13.2 m/s is higher than that of 10.2 m/s by 225.45% and 12 PM. Moreover, the GOR of the system at a seawater mass flow rate of 0.63 kg/s is higher than that of 0.39 kg/s by 68.9%.
- The maximum value of the total water productivity occurred at a seawater mass flow rate of 0.63 kg/s and *MR* of 0.44.
- The maximum value of the recovery ratio *RR* occurred at a seawater mass flow rate of 0.63 kg/s and *MR* of 0.44.
- The maximum value of the *GOR*_overall_ of 1.22 is obtained at a feed seawater mass flow rate of 0.63 kg/s and *MR* of 0.44. The *GOR*_overall_ value decreases from 1.22 to 0.34 as the mass flow rate ratio increases from 0.44 to 0.57.
- The estimated cost of distilled water production was approximately 0.017 $ per liter, while the CO_2_ emission was reduced by 6 tons annually due to solar energy usage.

## Data Availability

The datasets generated during and/or analyzed during the current study are available from the corresponding author upon reasonable request.

## List of symbols

*A*: Area (m^2^)
*CPL*: Cost per liter ($)
*C*_*P*_: Specific heat (kJ/kg K)
En_out_: Annual useful energy (kwh/year)
*F*: Fixed cost ($)
*h*_*fg*_: Latent heat of vaporization (kj/kg K)
*H*: Enthalpy (kj/kg)
*I*: Incident radiation (W/m^2^)
$$\dot{m}$$: Mass flow rate (kg/s)
*n*: The plate life time (year)
*P*: Power (kW)
*Q*: Heat absorbed by collector (kW)
*T*: Temperature (K)
*T*_∞_: Ambient temperature (K)
*V*: Velocity(m/s)

## Greek symbols

*η*: Efficiency (–)
$$\rho$$: Density (kg/m^3^)
ω: Absolute humidity (Kg/kg dry air)
*Ø*_co2_: Environmental factor (ton)

## Subscripts

*a*: Air
*c*: Collector
*d*: Dehumidifier
*dw*: Distilled water
*h*: Humidifier
*in*: Inlet
*p*: Pump
*rad*: Radiation
*sw*: Sea water

## Abbreviations

*GOR*: Gain output ratio
*MR*: Mass flow rate ratio
*RR*: Recovery ratio
*HDH*: Humidification–dehumidification
*CAOW*: Closed air open water
*PPT*: Part per thousand
